# Estradiol replacement improves high‐fat diet‐induced insulin resistance in ovariectomized rats

**DOI:** 10.14814/phy2.15193

**Published:** 2022-03-03

**Authors:** Naoko Yokota‐Nakagi, Sayo Omoto, Shoko Tazumi, Mizuho Kawakami, Akira Takamata, Keiko Morimoto

**Affiliations:** ^1^ Department of Environmental Health Faculty of Human Life and Environment Nara Women’s University Nara Japan; ^2^ Department of Health and Nutrition Faculty of Health Science Kyoto Koka Women’s University Kyoto Japan

**Keywords:** Akt2, AS160, estradiol replacement, high‐fat diet, insulin resistance, ovariectomized rat

## Abstract

The role of 17β‐estradiol (E2) in high‐fat diet (HFD)‐induced alteration of the protein kinase B (Akt) signaling pathway in ovariectomized (OVX) rats is unclear. Therefore, we examined whether chronic estrogen replacement restores HFD‐induced impairment in insulin sensitivity by its effects concomitant with alterations in the Akt isoform 2 (Akt2) and Akt substrate of 160 kDa (AS160) phosphorylation in muscles of OVX rats. Nine‐week‐old female Wistar rats underwent ovariectomy under anesthesia; after 4 weeks, subcutaneous implantation of either E2 or placebo (PL) pellets was performed, and HFD feeding was initiated. Intravenous glucose tolerance tests were performed to assess insulin sensitivity. Following insulin injection into rats’ portal vein, the liver and gastrocnemius muscle were dissected for insulin signaling analysis. We observed that HFD increased energy intake and body weight in the PL group; however, it was temporarily decreased in the E2 group. Adipose tissue accumulation was larger in HFD‐fed rats than in normal chow diet (NCD)‐fed rats in the PL group; however, this difference was not observed in the E2 group. HFD reduced insulin sensitivity in the PL group only. In vivo insulin stimulation increased Akt2 phosphorylation in the muscles of NCD‐fed rats in both groups. In contrast, HFD affected insulin‐stimulated phosphorylation of Akt2 and AS160 in the muscles of rats in the PL group but not in the E2 group. Our data suggest that E2 replacement improves HFD‐induced insulin resistance, and this effect is accompanied by the alterations in the Akt2 and AS160 phosphorylation in insulin‐stimulated muscles of OVX rats.

## Graphical abstract

This study examined whether chronic estrogen replacement restores high‐fat diet‐induced impairment in insulin sensitivity by its effects on insulin signaling pathway in ovariectomized rats. This is the first study to suggest that 17β‐estradiol replacement improves high‐fat diet‐induced insulin resistance, and this effect is accompanied by the alterations in the Akt2 and AS160 phosphorylation in insulin‐stimulated muscles of ovariectomized rats.

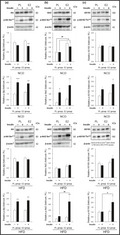

## 1 INTRODUCTION

Obesity and being overweight have been well established as major risk factors for type 2 diabetes, metabolic syndrome, and cardiovascular disease (Bray, 2004). It has been demonstrated that dietary fat intake results in weight gain and fat accumulation. Previous studies have shown that a high‐fat diet (HFD) (≥ 30% of calories from fat) easily induces obesity in humans (Hill et al., 2000; Schrauwen & Westerterp, 2000). Diets rich in fat have been shown to cause obesity in animals also (Boozer et al., 1995; Bourgeois et al., 1983; Ghibaudi et al., 2002; Hansson et al., 2018). Furthermore, HFD has been associated with impaired insulin‐mediated metabolic effects in humans and animals (Hansson et al., 2018; Kahn et al., 2006; Small et al., 2018; Woods et al., 2003). In a previous report, the results of a glucose tolerance test indicated that impaired glucose clearance was observed after 2 days of feeding on HFD, which gradually worsened with the number of days (14 days) of HFD feeding with 60% dietary fat in male mice (Hansson et al., 2018).

Impairment in the insulin signaling cascade has been suggested to result in insulin resistance (Petersen & Shulman, 2018; Yaribeygi et al., 2019). It is now well established that the binding of insulin to its receptor, insulin receptor substrate, and phosphatidylinositol (PI) 3‐kinase, causes phosphorylation and activation of protein kinase B (Akt) (Alessi & Downes, 1998; Saltiel & Kahn, 2001; Whiteman et al., 2002). Furthermore, Akt2, a highly expressed Akt isoform in skeletal muscle, is the key factor for insulin‐stimulated glucose uptake (Bouzakri et al., 2006; Cho et al., 2001; Gonzalez & McGraw, 2009). An Akt substrate of 160 kDa, AS160 (Rab‐GTPase activating protein), is phosphorylated at threonine (Thr)^642^ by Akt, and it regulates the trafficking of glucose transporter 4 (Chen et al., 2011; Sano et al., 2003).

Estrogen plays an important role in glucose homeostasis as well as body weight regulation in females (Mauvais‐Jarvis et al., 2013). Previous studies have demonstrated that estrogen withdrawal due to menopause or ovariectomy leads to body weight gain, visceral fat accumulation, and impaired insulin sensitivity in both humans and animals (Kawakami et al., 2018; Mauvais‐Jarvis et al., 2013; Munoz et al., 2002). Some previous studies on ovariectomized (OVX) rodents, which are widely used as models for investigating the pathophysiology of health problems in postmenopausal women, have reported the beneficial effects of estrogen on insulin sensitivity (Gorres et al., 2011; Kawakami et al., 2018; Moreno et al., 2010; Riant et al., 2009). Moreover, several studies reported that 17β‐estradiol (E2)‐replete female rodents were protected against HFD‐induced insulin resistance (Bryzgalova et al., 2008; Pratchayasakul et al., 2014; Riant et al., 2009), although insulin‐mediated glucose disposal was consistently reduced by 40–50% in male mice after HFD feeding (Choi et al., 2007; Hevener et al., 2007). Recently, we reported that the beneficial effects of E2 on insulin sensitivity were mediated by the activation of the Akt2/AS160 pathway in the muscles of OVX rats fed on a normal chow diet (NCD) (Kawakami et al., 2018). However, it remains unclear which components in the insulin signaling cascade are impaired by HFD and whether E2 improves the HFD‐induced impairment of the signaling pathway in OVX rats, although studies on male or intact female rodents have reported HFD‐induced impairment in Akt isoforms and their downstream signaling pathways (Badin et al., 2013; Stefanyk et al., 2011; Yaspelkis et al., 2007).

Therefore, the present study was designed to determine if chronic E2 replacement improves the HFD‐induced impairment in insulin sensitivity through the activation of the Akt2/AS160 pathway in the muscles of OVX rats. We assessed the effects of E2 replacement on whole‐body insulin sensitivity using an intravenous glucose tolerance test (IGTT) under free‐moving conditions and further analyzed in vivo insulin‐stimulated activation of the Akt2/AS160 pathway in the gastrocnemius muscle and liver of NCD‐ and HFD‐fed OVX rats.

## 2 MATERIALS AND METHODS

### 2.1 Animals and diets

All animal procedures conformed to the Standards relating to the Care and Keeping and Reducing Pain of Laboratory Animals (Notice of the Ministry of the Environment, Government of Japan) and ARRIVE guidelines and were approved by the Nara Women's University Committee on Animal Experiments (No. 17–02). Fifty‐three female Wistar rats were housed in individual cages containing paper bedding at 26 ± 1°C in a 12:12‐h light‐dark cycle (lights on at 07:00 AM). Access to food and water was provided ad libitum. All surgeries on rats were performed under anesthesia (pentobarbital sodium; 25–40 mg/kg intraperitoneally or isoflurane; 1.5–2.0% in oxygen).

Nine‐week‐old female rats fed using NCD (MF; Oriental Yeast, Tokyo, Japan) underwent ovariectomies. As previously described (Kawakami et al., 2018; Yokota‐Nakagi et al., 2020), after a 4‐week recovery period post‐ovariectomy, 13‐week‐old rats were randomly assigned to either the E2‐ or the placebo (PL)‐treated (*n* = 26 and *n* = 27, respectively) groups, and underwent subcutaneous implantation of either E2 (1.5 mg/60‐day release) or PL pellets (Innovative Research of America, Sarasota, FL, USA). Additionally, HFD (modified F2HFD2; Oriental Yeast) containing 498.9 kcal per 100 g (60.0% of calories from fat, predominantly lard) was initiated the day after the replacement. HFD and E2 replacement were continued until the end of the experiments for HFD‐fed rats (*n* = 12) in E2 and rats (*n* = 13) in PL groups. The NCD‐fed rats continued on NCD (*n* =14 in E2, *n* =14 in PL), which contained 360.0 kcal per 100 g (13.2% of calories from fat). The food intake and body weight of rats were measured daily during the experiments. The food intake was quantified by subtracting the amount measured the day before from the amount measured that day.

### 2.2 Assessment of insulin sensitivity using IGTT under free‐moving conditions

Rats aged 16 weeks in both the PL and E2 groups were implanted with intravenous catheters in the superior caval vein for IGTT. Four weeks after E2 and HFD replacement, the test was performed on 17‐week‐old rats using an unobstructed catheter (PL‐NCD, *n* = 7; PL‐HFD, *n* = 11; E2‐NCD, *n* = 12; E2‐HFD, *n* = 12) 5 days after cannulation, as previously described (Kawakami et al., 2018). Briefly, the rats were housed individually in experimental plastic cages and were acclimated to the experimental protocols using an extension tube for blood sampling the day before IGTT. Following 16 h of fasting and 1 h of rest after connecting the catheter with the extension tube, the blood sample (0.3 ml) was obtained from each catheter under free‐moving conditions and harvested in a plastic tube containing 50 mg/ml ethylenediaminetetraacetic acid. Subsequently, after glucose injection (1 g/kg), blood samples were collected at 5, 10, 30, and 60 min using a venous catheter. The red blood cells in saline were returned to the rats after each sample collection to prevent compensatory sympathoadrenal activation due to blood loss caused by the sampling. The collected blood samples were centrifuged, and the plasma was stored at −45°C until the analysis of plasma glucose or insulin concentration.

Plasma glucose and insulin concentrations were assayed by a glucose assay kit (Glucose CII‐Test kit, Wako Pure Chemical Industries, Osaka, Japan) and a rat insulin ELISA kit (AKRIN‐010T, Shibayagi, Gunma, Japan), respectively. Area under the curve (AUC) for analyzing the increase in plasma glucose and plasma insulin over the 60‐min period following the glucose injection was calculated using the trapezoidal method. The glucose‐insulin index was calculated as the product of the areas under the glucose and insulin curves following the glucose challenge to assess insulin resistance.

### 2.3 Sampling for estimation of insulin signaling analysis

The protocol for insulin signaling analysis was performed according to a previously described method (Kawakami et al., 2018). After a 16‐h fasting period, the 17‐week‐old rats (PL‐NCD, *n* = 12; PL‐HFD, *n* = 12; E2‐NCD, *n* = 11; E2‐HFD, *n* = 11) were anesthesized (isoflurane; 1.5–2.0% in oxygen). HFD and E2 replacement were maintained for 4 weeks before the tissue collections. After an abdominal incision, a single bolus of 10 ml/kg of physiological saline (0.9% NaCl) with or without 10^−5^ mol/l insulin (Novolin R 100 IU/ml; Novo Nordisk, Bagsværd, Denmark) was injected into the portal vein of each rat. The liver and gastrocnemius muscle were harvested 30 and 90 s, respectively, after the injection, according to the procedure described by Ogihara et al. (Ogihara et al., 2001). These tissues were then dissected, immediately frozen in liquid nitrogen, and stored at −50°C until insulin signaling analysis by western blotting.

When the experiments were completed, the rats were euthanized by overdosing with pentobarbital sodium and the wet weights of the mesenteric, kidney‐genital, retroperitoneal, and inguinal adipose tissues were measured. The total weight of the mesenteric, kidney‐genital, and retroperitoneal adipose tissues was calculated to determine the weight of visceral adipose tissue. The weight of the inguinal adipose tissue was evaluated as representative of that of the subcutaneous adipose tissue in each rat (Yokota‐Nakagi et al., 2020).

### 2.4 Western blotting

Protein levels from the liver and muscles were analyzed by western blotting according to previously published protocols (Kawakami et al., 2018; Yokota‐Nakagi et al., 2020).

The primary antibodies for Akt, phospho (p)‐Akt serine (Ser)^473^, Akt2, p‐Akt2 Ser^474^, and p‐AS160 Thr^642^ were purchased from Cell Signaling Technologies (Beverly, MA, USA). The AS160 antibody and the β‐actin antibody were obtained from Millipore (Temecula, CA, USA) and Sigma‐Aldrich (St. Louis, MO, USA), respectively. The dilutions of the antibodies were as follows: anti‐Akt (1:5000 or 1:3000), anti‐p‐Akt Ser^473^ (1:1000), anti‐Akt2 (1:1000), anti‐p‐Akt2 Ser^474^ (1:1000), anti‐AS160 (1:3000), anti‐p‐AS160 Thr^642^ (1:500), and anti‐β‐actin (1:2000). Goat anti‐rabbit horseradish peroxidase‐conjugated secondary antibody (1:7500 or 1:5000) was purchased from Promega (Madison, WI, USA) and used against all primary antibodies. The blots were developed with the imaging system Ez‐Capture and the image processing program CS Analyzer (ATTO, Tokyo, Japan) and normalized with the control β‐actin. In the analysis, protein levels (fold) were expressed as relative values calculated by dividing the value of the individual rat in each group by the average value for the PL‐NCD group injected saline. In this experiment, Akt2 or AS160 was not examined in the liver tissues by western blotting since previous reports have shown that the liver expresses low levels of AS160 (Hargett et al., 2016; Lansey et al., 2012).

### 2.5 Statistical analyses

Data were represented as means ± SE. Two‐way repeated‐measures ANOVA for each pair‐wise comparison among the four groups (PL‐NCD, PL‐HFD, E2‐NCD, and E2‐HFD), followed by a Tukey–Kramer post hoc test, was used to analyze the effect of E2 or HFD on the change in food intake, energy intake, body weight, and plasma glucose and insulin concentrations. Using two‐way factorial ANOVA followed by a Tukey–Kramer post hoc test, the effect of E2 on HFD‐induced changes in the wet weights of adipose tissues, glucose AUC, insulin AUC, glucose‐insulin index during IGTT, and comparison of the responses to insulin based on the levels of the insulin signaling proteins between the groups were analyzed. A *p*‐value < 0.05 was considered statistically significant.

## 3 RESULTS

### 3.1 Energy intake, body weight, and adipose tissue weight

**FIGURE 1:**
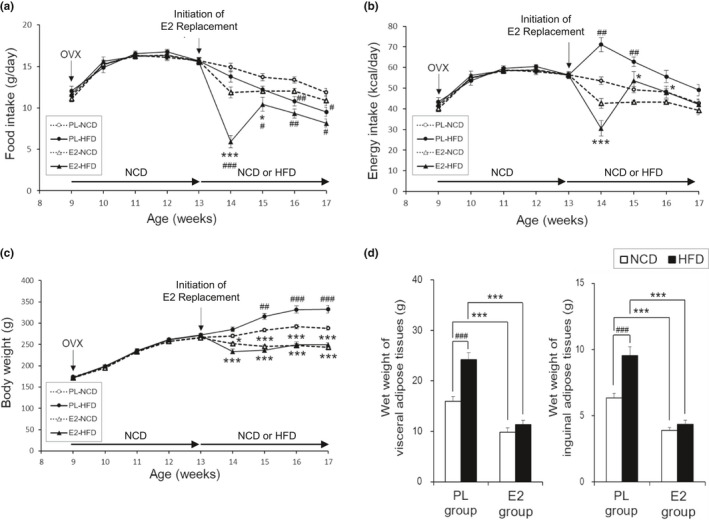
Changes in food intake (a), energy intake (b), body weight (c), and wet weights of adipose tissues (d) in ovariectomized (OVX) rats. Rats were assigned to the 17β‐estradiol (E2)‐replaced or placebo (PL)‐treated group after a 4‐week recovery period from the OVX operation. These rats were fed a normal chow diet (NCD) or a high‐fat diet (HFD). Values are means ± SE (*n* = 14 in PL‐NCD; *n* = 13 in PL‐HFD; *n* = 14 in E2‐NCD; *n* = 12 in E2‐HFD). There was an interaction of age or diet and group effects in food intake (*p* < 0.05: PL‐NCD vs. PL‐HFD; *p* < 0.001: PL‐HFD vs. E2‐HFD, E2‐NCD vs. E2‐HFD), energy intake (*p* < 0.01: PL‐NCD vs. PL‐HFD; *p* < 0.001: PL‐HFD vs. E2‐HFD), body weight (*p* < 0.01: E2‐NCD vs. E2‐HFD; *p* < 0.001: PL‐NCD vs. PL‐HFD, PL‐NCD vs. E2‐NCD, PL‐HFD vs. E2‐HFD), and the wet weights of visceral (*p* < 0.01) and inguinal adipose tissues (*p* < 0.01). * and ***, significant differences (*p* < 0.05 and *p* < 0.001, respectively) between the PL and E2 groups. ^#^, ^##^, and ^###^, significant differences (*p* < 0.05, *p* < 0.01, and *p* < 0.001, respectively) between the NCD and HFD groups

HFD had no effect on food intake in rats aged 14–15 weeks in the PL group; however, HFD‐fed rats demonstrated a gradually decreased food intake at 16–17 weeks when compared with that of the NCD‐fed rats (Figure 1a). In contrast, HFD‐fed rats demonstrated a decreased food intake at 14–17 weeks when compared to NCD‐fed rats in the E2 group. Therefore, HFD did not affect energy intake in rats in the E2 group; however, it was enhanced in rats aged 14–15 weeks in the PL group, resulting in a difference in energy intake between the PL and E2 groups of only HFD‐fed rats (Figure 1b). Rats aged 15–17 weeks, when fed on HFD, showed higher body weights than those fed on NCD in the PL group. However, this difference was not observed in the E2 group, resulting in a difference in body weight between the PL and E2 groups (Figure 1c). Statistical analysis showed an association between age and group effects based on food intake (*p* < 0.05: PL‐NCD vs. PL‐HFD; *p* < 0.001: PL‐HFD vs. E2‐HFD, E2‐NCD vs. E2‐HFD), energy intake (*p* < 0.01: PL‐NCD vs. PL‐HFD; *p* < 0.001: PL‐HFD vs. E2‐HFD), and body weight (*p* < 0.01: E2‐NCD vs. E2‐HFD; *p* < 0.001: PL‐NCD vs. PL‐HFD, PL‐NCD vs. E2‐NCD, PL‐HFD vs. E2‐HFD).

HFD‐fed rats showed significantly increased wet weights of total visceral and inguinal subcutaneous adipose tissues (Figure 1d) in the PL group; however, this difference was not observed in the E2 group. There was an association between diet and group effects based on wet weights of visceral (*p* < 0.01) and inguinal (*p* < 0.01) adipose tissues. Therefore, E2 replacement suppressed HFD‐induced body weight gain and fat accumulation by regulating the energy intake in OVX rats.

### 3.2 Assessments of insulin sensitivity by IGTT using free‐moving rats

**FIGURE 2:**
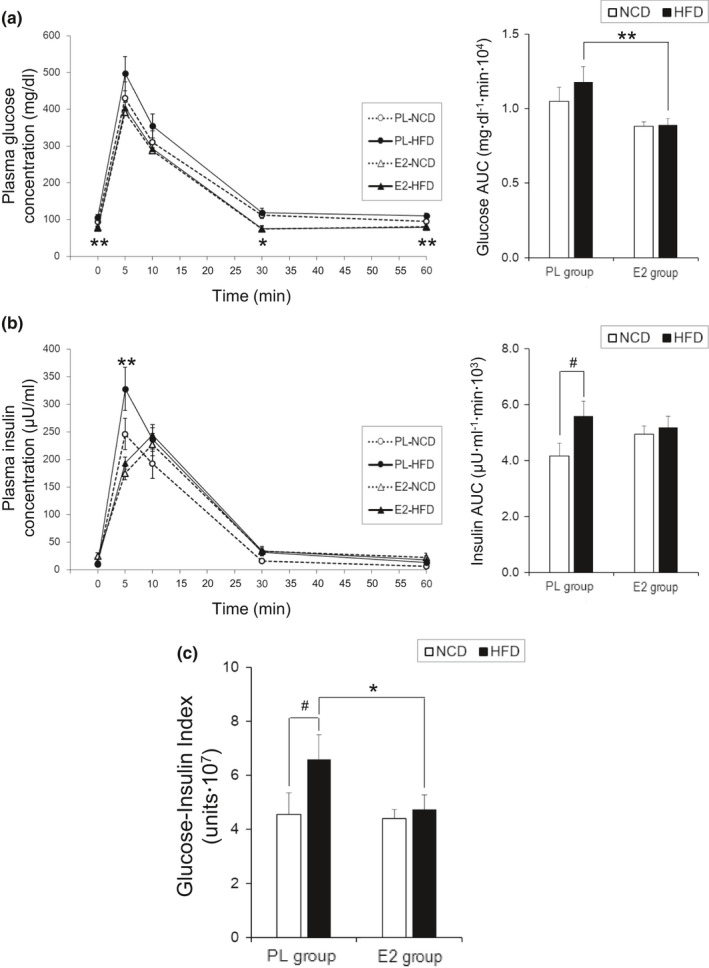
Assessments of insulin sensitivity by the intravenous glucose tolerance test in ovariectomized rats. Values are means ± SE [*n* =7 in placebo (PL)‐normal chow diet (NCD); *n* = 11 in PL‐ high‐fat diet (HFD); *n* = 12 in 17β‐estradiol (E2)‐NCD; *n* = 12 in E2‐HFD]. Line graphs represent the courses of change in plasma glucose (Left side of a) and insulin (Left side of b) concentration after glucose injection in the four groups. There was an interaction of time and group effects in the plasma insulin response (*p* < 0.05: PL‐NCD vs. E2‐NCD; *p* < 0.01: PL‐HFD vs. E2‐HFD), but not in the plasma glucose response. Bar graphs represent the area under the curve (AUC) for glucose (Right side of a) and insulin (Right side of b) levels over 60 min after the glucose injection and glucose‐insulin index (c) in each group. * and **, significant differences (*p* < 0.05 and *p* < 0.01, respectively) between the PL and E2 groups. ^#^, significant differences (*p* < 0.05) between the NCD and HFD groups

Fasting glucose levels were higher in HFD‐fed rats of the PL group than in those of the E2 group (*p* < 0.01). Following glucose injections, plasma glucose concentrations rapidly increased; it reached peak levels at 5 min and then gradually returned to basal levels over a 30‐min period. Glucose levels at 5 and 10 min did not differ between the groups, although significant differences were observed at 30 and 60 min in HFD‐fed rats of the PL and E2 groups (*p* < 0.05 and *p* < 0.01, respectively). Moreover, no association between time and group effects was detected. E2 replacement and HFD had no effect on the glucose AUC in NCD‐fed OVX rats; however, E2 attenuated the AUC in HFD‐fed OVX rats (*p* < 0.01) (Figure 2a).

As shown in Figure 2b, plasma insulin levels increased rapidly after glucose injections and showed peak values at 5 min in the PL group and at 10 min in the E2 group. There was a significant difference in plasma insulin levels at 5 min between the two HFD‐fed groups (*p* < 0.01). The insulin response over 60 min was significantly different between the PL and E2 groups regardless of the type of diet (interaction, *p* < 0.05: PL‐NCD vs. E2‐NCD and *p* < 0.01: PL‐HFD vs. E2‐HFD). Additionally, HFD augmented the insulin AUC in the PL group (*p* < 0.05) but not in the E2 group (Figure 2b).

As shown in Figure 2c, the glucose‐insulin index established for the analysis of insulin resistance index was observed to be enhanced in HFD‐fed rats when compared with NCD‐fed rats only in the PL group (*p* < 0.05). Moreover, the HFD‐fed rats in the PL group showed a higher index than those in the E2 group (*p* < 0.05).

### 3.3 In vivo effect of E2 replacement on insulin signaling

**FIGURE 3:**
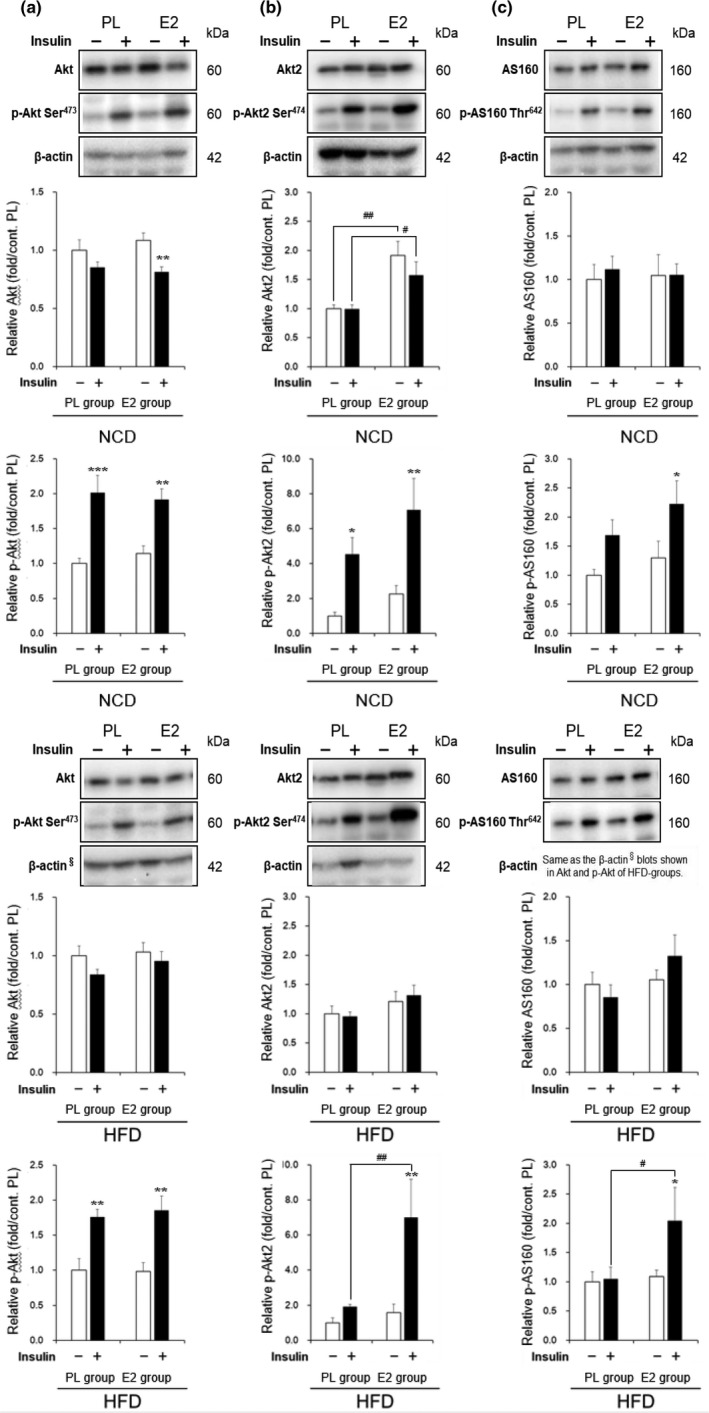
Effects of high‐fat diet (HFD) and 17β‐estradiol (E2) replacement on insulin‐stimulated protein kinase B (Akt)/Akt substrate of 160 kDa (AS160) pathway in muscles. Representative blots and relative values of Akt and phospho (p)‐Akt serine (Ser)^473^ (a), Akt2 and p‐Akt2 Ser^474^ (b), and AS160 and p‐AS160 threonine (Thr)^642^ (c) in the gastrocnemii of ovariectomized rats. Protein levels were normalized with the control β‐actin and were expressed as relative values calculated by dividing the value of the individual rat in each group by the average value for the placebo (PL)‐normal chow diet (NCD) group injected saline. Values are means ± SE, with (+) or without (–) insulin stimulation by injection of 10^−5^ mol/l insulin or saline in the portal vein [*n* =12 in PL‐NCD; *n* = 12 in PL‐HFD; *n* = 11 in E2‐NCD; *n* = 11 in E2‐HFD]. There was an interaction of replacement and insulin injection effects in the insulin‐induced phosphorylation of Akt2 Ser^474^ in the HFD‐fed rats (*p* < 0.05). *, **, and ***, significant differences (*p* < 0.05, *p* < 0.01, and *p* < 0.001, respectively) between saline and insulin injections. ^#^ and ^##^, significant differences (*p* < 0.05 and *p* < 0.01, respectively) between the PL and E2 groups

**FIGURE 4:**
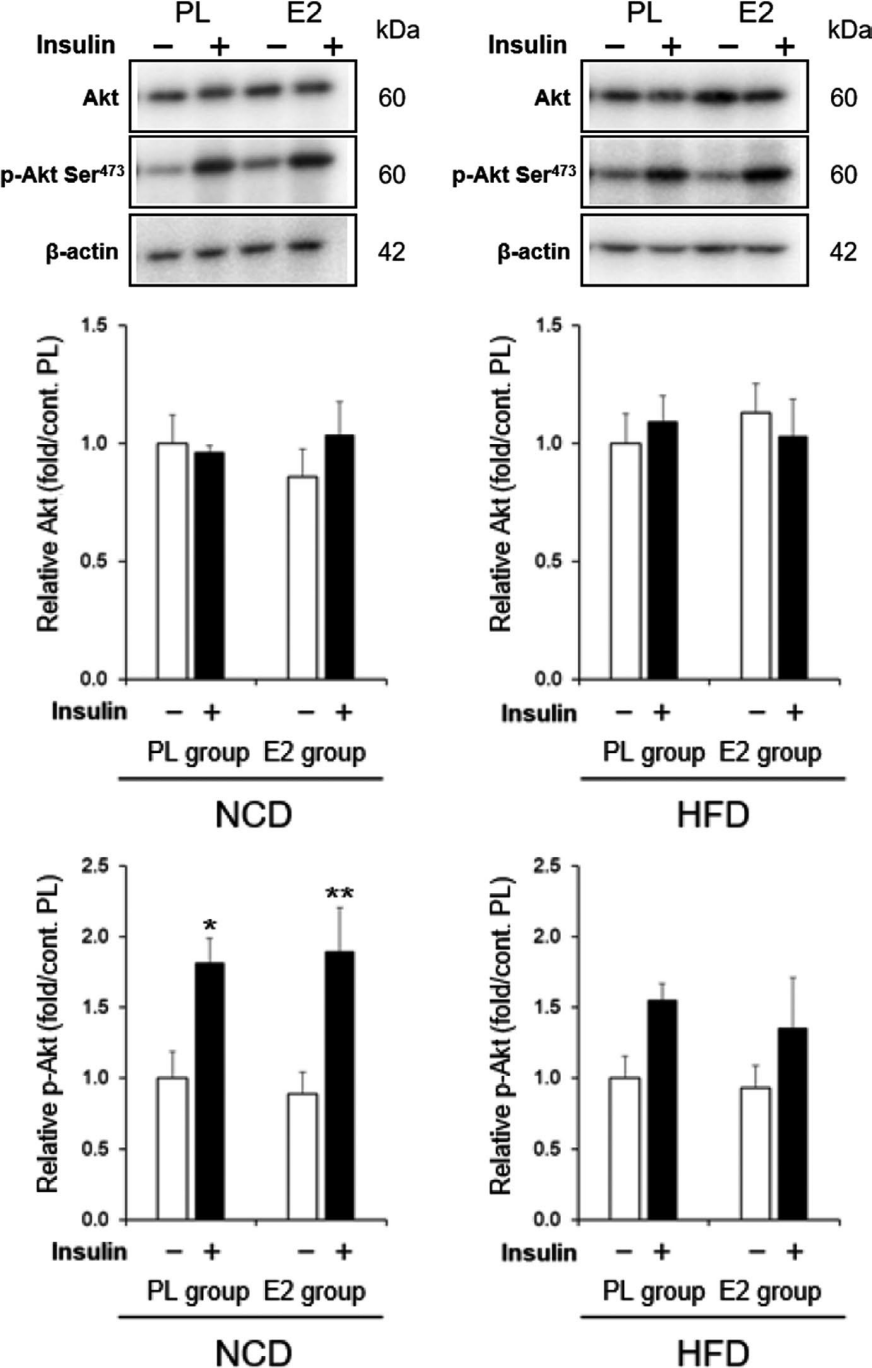
Effects of high‐fat diet (HFD) and 17β‐estradiol (E2) replacement on insulin‐stimulated protein kinase B (Akt) activation in livers. Representative blots and relative values of Akt and phospho (p)‐Akt serine (Ser)^473^ in the livers of ovariectomized rats. Protein levels were normalized with the control β‐actin and were expressed as relative values calculated by dividing the value of the individual rat in each group by the average value for the placebo (PL)‐normal chow diet (NCD) group injected saline. Values are means ± SE, with (+) or without (–) insulin stimulation by injection of 10^−5^ mol/l insulin or saline in the portal vein [*n* = 12 in PL‐NCD; *n* = 12 in PL‐HFD; *n* = 11 in E2‐NCD; *n* = 11 in E2‐HFD]. * and **, significant differences (*p* < 0.05 and *p* < 0.01, respectively) between saline and insulin injections

To elucidate the molecular mechanism underlying the beneficial effect of E2 on insulin sensitivity in HFD‐fed OVX rats, we examined the key proteins involved in the insulin signaling cascade in the gastrocnemius muscle (Figure 3) and liver (Figure 4).

As shown in Figure 3a, the levels of Akt protein in the non‐stimulated muscles were similar in the PL and E2 groups, regardless of whether they were fed on NCD or HFD. Insulin injection increased phosphorylation of Akt Ser^473^ in the muscles of NCD‐ and HFD‐fed rats to a similar extent in the PL and E2 groups. Figure 3b shows that Akt2 protein levels were higher in the E2 group than in the PL group with NCD‐fed rats. Insulin stimulated phosphorylation of Akt2 Ser^474^ in the muscles of NCD‐fed rats in the PL and E2 groups. In contrast to NCD‐fed rats, E2 had no effect on Akt2 protein level in HFD‐fed rats; however, it increased p‐Akt2 level in insulin‐stimulated muscles (*p* < 0.01: PL‐HFD vs. E2‐HFD). An association between insulin and group effects was observed in the insulin‐stimulated phosphorylation of Akt2 Ser^474^ in HFD‐fed rats (*p* < 0.05). Figure 3c shows that insulin enhanced the phosphorylation of AS160 Thr^642^ in the muscles of rats in the E2 group only, regardless of whether they were fed on NCD or HFD (*p* < 0.05). Additionally, HFD‐fed rats in the E2 group showed higher levels of phosphorylated AS160 than in the PL group (*p* < 0.05).

Figure 4 shows Akt and p‐Akt Ser^473^ protein levels in the liver of NCD‐ and HFD‐fed rats in the PL and E2 groups. Insulin injection stimulated Akt phosphorylation in the two groups fed on NCD. However, the insulin‐induced phosphorylation was no longer statistically significant in HFD‐fed rats in both groups.

## 4 DISCUSSION

In the present study, we demonstrated that E2 replacement restored HFD‐induced impairment in insulin sensitivity by activating the insulin‐signaling pathway in the muscles of OVX rats. To our knowledge, this is the first study to demonstrate HFD‐induced impairment and E2‐induced positive impact of the in vivo insulin‐stimulated Akt2/AS160 pathway in HFD‐fed OVX rats.

Menopause is associated with increased metabolic syndrome or type 2 diabetes (Munoz et al., 2002). The percentage of energy intake from fat increases significantly in the postmenopausal years (Lovejoy et al., 2008). In this study, the postmenopausal HFD‐induced obese rat model has greater relevance with respect to women, as overconsumption of calories after menopause leads to increased fat mass and insulin resistance, in comparison with genetically obese rodent models (Xu et al., 2003).

A previous study has also reported the protective effect of E2 treatment on HFD‐induced insulin resistance, which was evaluated based on a simple homeostatic model assessment index in OVX rats (Pratchayasakul et al., 2014). However, there is increasing evidence that E2 improves HFD‐induced insulin resistance in OVX mice (Camporez et al., 2013; Riant et al., 2009). Additionally, the effect of E2 on insulin sensitivity was reported to be dose‐dependent since an excess or a lack of E2 was observed to be associated with insulin resistance in OVX rats (Gonzalez et al., 2003).

In this study, the rats in the E2 group spent 4 weeks in E2‐replete condition after 4 weeks in an E2‐deficient state; hence, they were used as postmenopausal models that underwent E2 replacement therapy. However, there was a difference in aging as well as between the postmenopausal model rats and naturally menopausal women because the rats underwent ovariectomy at 13‐week ages. The plasma E2 concentrations were not measured in this study but may be consistent with that of our previous study, with 44.1 ± 10.3 pg/ml in the E2 group and 9.2 ± 0.5 pg/ml in the PL group using the same pellets as that of the present study (Kawakami et al., 2018). Usually, E2 replacement is performed based on plasma E2 levels in intact female rats. The rats in the E2 group had sustained high levels of E2, within the accepted range for intact female rats in proestrus, close to the levels reported in previous studies (Asarian & Geary, 2013). Furthermore, the previous study reported that HFD feeding had no effects on plasma E2 levels (Pratchayasakul et al., 2014). The finding supports that plasma E2 in this study may be similar to our previous study using rats fed NCD. Therefore, the present results provide in vivo data to support the idea that E2 replacement restores insulin sensitivity impaired by HFD in postmenopausal women, though it should be prudent to apply the results directly to the women.

This study demonstrated that HFD for 4 weeks with 60% energy from fat decreased insulin sensitivity in E2‐depleted rats. The composition and fat content of HFD and the duration of HFD feeding influenced the effects of HFD on insulin sensitivity. HFD, generally used in studies on rodents, contains the amount of dietary fat that is 45–60% of their total energy intake (Buettner et al., 2007; Small et al., 2018). Furthermore, some studies have reported that different fatty acid (FA) contents in dietary fat lead to different metabolic outcomes in HFD‐fed rats (Buettner et al., 2006, 2007; Storlien et al., 1991). In rats, saturated FA‐rich diets were reported to cause a greater degree of insulin resistance than polyunsaturated FA‐rich diets (Buettner et al., 2006; Storlien et al., 1991). Our results are consistent with these previous findings because the HFD used in this study contained predominantly saturated and monounsaturated FAs (24% energy saturated; 27% energy monounsaturated; 8% energy polyunsaturated). Furthermore, a previous study reported that whole‐body insulin resistance was rapidly detected 1 week after the initiation of HFD due to hepatic insulin resistance, while skeletal muscle displayed insulin resistance at 3 weeks in male mice (Turner et al., 2013). However, another study found that insulin resistance in skeletal muscle, adipose tissue, and liver occurred simultaneously after 3 weeks of HFD‐feeding in male mice (Park et al., 2005). These findings show that 4 weeks of HFD‐feeding in this study may be a sufficient duration for rodents to develop insulin resistance.

In this study, the gastrocnemius muscle was examined to elucidate the mechanism of estrogen action, because this muscle predominantly composed of fast‐twitch (type II) fibers, was widely used to evaluate the insulin signaling pathway resulting in glucose uptake (Baum et al., 2005; Ndisang et al., 2010). Sufficient activation of the PI 3‐kinase/Akt pathway is essential for insulin‐stimulated glucose uptake by the skeletal muscles. Based on recent evidence, insulin‐stimulated phosphorylation of AS160 at the Thr^642^ and Ser^588^, downstream of the PI 3‐kinase/Akt pathway, is an important mechanism for increased glucose transporter 4 translocation and glucose transport in the muscles (Sakamoto & Holman, 2008; Sano et al., 2003). Therefore, our study indicated for the first time that HFD impaired insulin‐stimulated phosphorylation of Akt2 Ser^474^ in the gastrocnemius muscle of OVX rats and that E2 replacement restored the phosphorylation of Akt2 Ser^474^ and increased the p‐AS160 Thr^642^. In summary, E2 replacement may maintain glucose uptake in the muscles by sustaining insulin‐stimulated phosphorylation in the Akt2/AS160 pathway under HFD‐feeding conditions in OVX rats.

Our data also showed that 4 weeks of HFD‐feeding inhibited insulin‐stimulated Akt activation in the liver and that E2 replacement did not restore the HFD‐induced impairment in the liver, whereas it improved the activation of the Akt2/AS160 pathway in the muscles. Distinct patterns of tissue‐specific E2 effects on HFD‐induced impairment may be related to differing onsets and duration of insulin resistance in the liver and muscles, as previously reported (Turner et al., 2013).

The link between lipid oversupply and insulin resistance is well investigated, and evidence on the mechanisms by which lipids cause the development of muscle insulin resistance has been reported (Schmitz‐Peiffer, 2000). When the amount of lipids in the blood chronically exceeds the uptake and storage capacity of white adipose tissue, FAs accumulate in other tissues, such as the liver and skeletal muscles. In the muscles, FAs accumulate intracellularly in myocytes, primarily as long‐chain fatty acyl‐CoA (Cooney et al., 2002, Schmitz‐Peiffer, 2000). Among the FA derivatives, high levels of diacylglycerol and ceramides in the myocytes are directly associated with insulin resistance in the muscles (Schmitz‐Peiffer, 2000). Notably, increased intracellular levels of diacylglycerol in the muscles have been shown to activate the signaling of protein kinase C, resulting in Ser phosphorylation of insulin receptor substrate‐1. It inhibits the phosphorylation of tyrosine residues on insulin receptor substrate‐1, leading to a defect in the canonical PI 3‐kinase/Akt signaling pathway and consequently reducing insulin‐stimulated glucose uptake in the muscles (Schmitz‐Peiffer, 2000). A similar mechanism may exist in the present study. In HFD‐fed OVX mice, a recent study showed that E2 replacement reduced diacylglycerol content in both the liver and muscles, decreased protein kinase C activation, and promoted insulin‐stimulated Akt2 phosphorylation (Camporez et al., 2013).

In the context of this study, it is plausible that the effect of E2 replacement on insulin resistance in HFD‐fed OVX rats is directly mediated by estrogen receptors (ERs). Additionally, the effects of E2 on insulin sensitivity may be mediated at least partly by E2‐induced inhibition of abdominal obesity. To directly assess the anorexigenic effect of E2 replacement on glucose homeostasis, a pair‐fed group would be included in our experiments. Our previous study, which used NCD‐fed rats, showed that food restriction by pair‐feeding to E2 replacement group in the PL group ameliorated hyperglycemia in the OVX rats but failed to mimic the effects of E2 replacement on basal levels of insulin signaling components, Akt2 and AS160 (Kawakami et al., 2019). This finding supports direct E2 action of the signaling pathway instead of anorexia‐induced indirect action. Therefore, even under the HFD conditions in the present study, E2 may directly affect insulin sensitivity and signaling pathways. Previously, Riant et al. showed that in HFD‐fed female mice, the beneficial effects of E2 on insulin sensitivity were mediated through ERα activation because this action was abolished in ER‐deficient mice (Riant et al., 2009). This study showed for the first time that HFD‐induced impairment of the insulin signaling pathway and E2‐induced improvement of the in vivo insulin‐stimulated Akt2/AS160 pathway in HFD‐fed OVX rats. Only one previous report using NCD‐fed OVX rats has shown that an ERα agonist enhanced insulin‐stimulated glucose uptake into the skeletal muscles by increasing insulin‐stimulated phosphorylation of Akt and increasing glucose transporter 4 protein but not insulin‐stimulated phosphorylation of AS160 (Gorres et al., 2011). ERα and ERβ are expressed in rodent and human skeletal muscles (Gorres et al., 2011; Lemoine et al., 2003; Wiik et al., 2003); therefore, it is likely that ERs are involved in E2‐induced activation of the Akt2/AS160 pathway.

In this study, E2 replacement suppressed the increase in energy intake and body weight in HFD‐fed OVX rats more strongly than in NCD‐fed rats, as already shown in our previous report (Yokota‐Nakagi et al., 2020). Contrarily, a previous study reported that food intake was slightly lower in OVX mice than E2‐replaced mice fed on HFD, despite being more obese (Camporez et al., 2013). Furthermore, another study showed that E2‐replaced mice and OVX mice demonstrated similar body weights when fed on HFD for 1 month (Riant et al., 2009). Additionally, a previous study using OVX Sprague‐Dawley rats reported that those fed on HFD demonstrated similar body weights and daily energy intake when compared with those fed on NCD (Paquette et al., 2007). These discrepancies may be attributed to the difference in rodent species and the strain of rats. This idea is supported by a study showing that the metabolic effects caused by HFD seemed to be more pronounced in Wistar rats than in Sprague‐Dawley rats (Marques et al., 2016).

Our results suggest that postmenopausal women should refrain from HFD and properly adjust their dietary fat intake to prevent insulin resistance. However, we acknowledge that there are several limitations to the present study. First, the present study does not reveal how HFD causes impairment of insulin signaling in the muscles of OVX rats. Second, the results of IGTT and insulin signaling cannot be compared because there may be a difference in plasma insulin concentrations between the tissue sampling experiment for insulin signaling and the IGTT experiment. In the tissue sampling, the plasma insulin levels at sampling points might be higher than the physiological insulin concentrations and change rapidly. Though insulin levels were not measured in this study or the previous literature, the experimental condition might induce animal‐to‐animal variability in plasma insulin levels. Third, this study does not provide data showing that ERs are directly involved in the activation of the Akt2/AS160 pathway. Further studies are required to elucidate the mechanisms accounting for HFD‐induced insulin resistance, which may be improved by E2 via ERs, as demonstrated in our rat model. Moreover, it is necessary to clarify the effects of E2 replacement and HFD on glucose uptake by muscle and hepatic glucose production.

In conclusion, this in vivo study suggests that E2 replacement improves HFD‐induced deterioration of whole‐body insulin sensitivity by activating the Akt2/AS160 pathway in insulin‐stimulated muscles of OVX rats. Our results also suggest the potential merit of E2 replacement for the possible prevention of HFD‐induced insulin resistance in postmenopausal women, though it should be prudent to apply the effects directly to the women.

## CONFLICT OF INTEREST

The authors declare that the research was conducted in the absence of any commercial or financial relationships that could be construed as a potential conflict of interest.

## AUTHOR CONTRIBUTIONS

Conceptualization, N.Y.‐N. and K.M.; Methodology, N.Y.‐N., A.T., and K.M.; Validation, N.Y.‐N., S.O., S.T., M.K, and K.M.; Formal analysis, N.Y.‐N., and K.M.; Investigation, N.Y.‐N., S.O., S.T., M.K., and K.M.; Writing—original draft preparation, N.Y.‐N. and K.M.; Writing—review and editing, N.Y.‐N., A.T., and K.M.; Supervision, K.M.; Project administration, N.Y.‐N. and K.M.; Funding acquisition, N.Y.‐N. and K.M. All authors have read and agreed to the published version of the manuscript.
